# Recovery of Adrenal Function after Long-Term Glucocorticoid Therapy for Giant Cell Arteritis: A Cohort Study

**DOI:** 10.1371/journal.pone.0068713

**Published:** 2013-07-24

**Authors:** Yvan Jamilloux, Eric Liozon, Gregory Pugnet, Sylvie Nadalon, Kim Heang Ly, Stephanie Dumonteil, Guillaume Gondran, Anne-Laure Fauchais, Elisabeth Vidal

**Affiliations:** 1 Department of Internal Medicine, Limoges University Hospital, Limoges, France; 2 Department of Internal Medicine, Hopital Purpan, Université de Toulouse, Toulouse, France; University of Patras Medical School, Greece

## Abstract

**Objectives:**

Giant cell arteritis (GCA) is a chronic systemic vasculitis of large and medium-sized arteries, for which long-term glucocorticoid (GC) treatment is needed. During GC withdrawal patients can suffer adrenal insufficiency. We sought to determine the time until recovery of adrenal function after long-term GC therapy, and to assess the prevalence and predictors for secondary adrenal insufficiency.

**Subjects and Design:**

150 patients meeting the ACR criteria for GCA between 1984 and 2012 were analyzed. All received the same GC treatment protocol. The low-dose ACTH stimulation test was repeated annually until adrenal recovery. Biographical, clinical and laboratory data were collected prospectively and compared.

**Results:**

At the first ACTH test, 74 (49%) patients were non-responders: of these, the mean time until recovery of adrenal function was 14 months (max: 51 months). A normal test response occurred within 36 months in 85% of patients. However, adrenal function never recovered in 5% of patients. GC of >15 mg/day at 6 months, GC of >9.5 mg/day at 12 months, treatment duration of >19 months, a cumulative GC dose of >8.5 g, and a basal cortisol concentration of <386 nmol/L were all statistically associated with a negative response in the first ACTH test (*p* <0.05).

**Conclusion:**

Adrenal insufficiency in patients with GCA, treated long-term with GC, was frequent but transitory. Thus, physicians’ vigilance should be increased and an ACTH test should be performed when GC causes the above associated statistical factors.

## Introduction

Giant cell arteritis (GCA) is a chronic systemic vasculitis of large and medium-sized arteries. It usually affects people aged >50 years, and affects an estimated 2/1000 individuals [1]. High-dose and long-term glucocorticoids (GC) are the mainstay treatment [2]. Despite wide inter-individual variability, a majority of patients can discontinue GC after 1–2 years [1].

Although chronic inflammation is known to inhibit the hypothalamic-pituitary-adrenal (HPA) axis [3], glucocorticoid therapy remains the first cause of HPA dysfunction [4]. This inhibition occurs when GC are given at a high-physiological dosage and/or for a prolonged period: it can sometimes lead to slow adrenal insufficiency or even an adrenal crisis [5]. The prevalence of HPA dysfunction has been reported in 15–100% of GC-treated patients [6]: this wide range is presumably due to the variety of patients, diseases, and GC regimens [6].

Testing the responsiveness of the HPA axis can be useful when prednisone dosage reaches ≤5 mg/day. For this purpose, three tests are available: the ACTH stimulation test, the metyrapone test and the insulin-induced hypoglycaemia test. The last two explore the entire HPA axis. Nevertheless, the low-dose ACTH stimulation test is probably the most convenient option to explore adrenal dysfunction after GC treatment [7].

A positive ACTH test enables weaning from GC, whereas a negative response can lead to hormone substitution. The mean time until a responsive HPA-range is obtained is 1–18 months after GC discontinuation [8,9]. To affirm definitive HPA inhibition, most authors recommend exploring the entire HPA axis [7]. However, to date, no study has evaluated the need for repeating the ACTH test after long-term GC treatment of GCA.

In the present study, we sought to determine the time until recovery of the normal adrenal response after GC treatment in a cohort of GCA patients. We also analysed the prevalence and predictive factors for GC-induced adrenal insufficiency.

## Methods

### Patients and data collection

Consecutive patients diagnosed with GCA, who met the American College of Rheumatology criteria, entered our prospective cohort study between 1984 and 2009. The study took place in the department of internal medicine, University Hospital of Limoges, France. All the patients gave their informed, written consent.

Using a comprehensive questionnaire, a senior internist recorded baseline clinical and laboratory parameters, and also prospectively assessed the outcome data (Table 1).

**Table 1:** Baseline characteristics, outcomes and comparisons between responders and non-responders to the first ACTH stimulation test during glucocorticoid (GC) treatment for giant cell arteritis (GCA).

**Characteristics**	**Total**	**Responders (*n*=76)**	**Non-responders (*n*=74)**	***P***
**Median age at diagnosis of GCA (years)**	74 ± 7	74 ± 7.5	74 ± 7.2	0.7
**Gender (male/female)**	49/101	20/56	29/45	0.09
**Median weight at GCA diagnosis (kg)**	62 ± 12	61 ± 13	63.5 ± 12	0.16
**Treatment**				
Starting dosage of GCs (mg/day)	51 ± 16	50 ± 15	52 ± 16	0.28
Initial pulse methylprednisolone (%)	25	31	19	0.1
GC dosage after 3 months (mg/day)	19 ± 5	17.6 ± 4.9	20.1 ± 5.6	0.003
GC dosage after 6 months (mg/day)	13 ± 4	11.9 ± 3.3	14.7 ± 4.8	0.0003
GC dosage after 12 months (mg/day)	8 ± 4	6.63 ± 3.2	8.9 ± 4.4	0.0007
Time from diagnosis to the first ACTH test (months)	17 ± 8	15.7 ± 7	18.6 ± 9	0.004
Total amount of GCs at the first ACTH test (mg)	7740 ± 3390	6800 ± 2729	8697 ± 3743	0.0007
**Basal cortisol concentration (nmol/L)**	392 ± 163	488 ± 121	295 ± 143	< 0.0001
**Stimulated cortisol concentration (nmol/L)**	566 ±212	728 ± 135	397 ± 124	< 0.0001
**Initial presentation**				
Symptomology (%)	75	80	69	0.16
Fatigue (%)	67	74	60	0.07
Fever (%)	43	42	47	0.6
Associated PMR (%)	32	33	31	0.8
ESR (mm)	87 ± 27	87.7 ± 29.3	86.3 ± 25.1	0.8
CRP (mg/mL)	97 ± 58	96.9 ± 59.4	98 ± 56.7	0.7
Haemoglobin (g/dL)	11.5 ± 1.8	11.4 ± 1.8	11.6 ± 1.7	0.6
**Evolution**				
Relapse (%)	50	52	48	0.7
Recurrence (%)	33	37	28	0.4
**Other GC-induced adverse events (%)**	60	63	59	0.7
Diabetes mellitus (%)	15	15	16	0.8
Myopathy (%)	25	24	27	0.7
Bone involvement (%)	15	15	15	1
Infection (%)	26	26	27	0.8

ESR: erythrocyte sedimentation rate; CRP: C-reactive protein; PMR: polymyalgia rheumatica

### Ethics approval

This study was conducted with the approval of the ethics committee of Limoges University Hospital, France.

### Treatment

All patients were treated according to the following standardized GC protocol. Patients without ischemic symptoms received 0.7 mg/kg/day of prednisone until the patient was symptom-free and the C-reactive protein concentration had fallen to <5 mg/L. Prednisone dosage was then progressively tapered to 0.35 mg/kg/day within 4 to 6 weeks, and then more slowly. Patients with ischemic symptoms received prednisone at 1 mg/kg/day, often preceded by pulsed methylprednisolone, and were then tapered similarly. The slow tapering protocol was as follows: reduction of 10 mg/day every 2 weeks until a dosage of 30 mg/day, then reduction of 5 mg/day every 2-3 weeks until a dosage of 20 mg/day, then reduction of 2.5 m/day every 15-21 days until a dosage of 10 mg/day, and then a reduction of 1 mg/day every month until a dosage of 5 mg/day. Physicians were given the opportunity to modulate the slow tapering phase according to their appreciation of the clinical situation. Below a prednisone dosage of 5 mg/day, GC tapering was continued, but patients with a negative response to ACTH stimulation also received hormone substitution.

### ACTH stimulation test

When prednisone dosage could be reduced to 5 mg/day, the low-dose ACTH stimulation test was performed according to the previously described procedure [10]. Briefly, after an overnight fast, prednisone intake was skipped and an intravenous catheter was inserted. Baseline cortisol level was measured at 8: 00 am and 1 µg of tetracosactide (Synacthen^®^, Sigma-Tau, France) was injected. Samples to detect cortisol dosage were collected after 30 and 60 min. Analyses for cortisol were performed in our hospital laboratory using radio-immuno-competition on transcortin and the Murphy technique [10]. This technique has low cross-reactivity with other glucocorticoids. A cortisol concentration of ≥580 nmol/L was considered to be a normal response, regardless of the basal cortisol level. Patients with a cortisol concentration of <580 nmol/L were considered to be non-responders and were re-tested annually until recovery.

### Statistical analyses

We conducted the data analyses with XLSTAT^©^ software, version Mac 2012 (Addinsoft, Paris, France). The means from continuously distributed data were tested for statistical differences between groups using the two-tailed Mann–Whitney U-test.

To transfer continuous data into discrete units, we determined the cut-off points for GC dosage at different end-points (3, 6, 12 months), for basal cortisol level, treatment duration and cumulative GC dose, using the nested-average method. For each parameter, the cut-off points were taken from the intersection of the curves between the responders and non-responders. This method gave more accurate thresholds than using median values.

Multivariate analyses (logistic regression) were performed on variables that reached a 25% significance level in univariate analysis; *p*<0.05 was considered statistically significant.

## Results

### Patients’ characteristics

A total of 150 consecutive patients (mean age 74±7 years; 101 females [67%]) diagnosed with GCA (106 biopsy proven [71%]) had at least one ACTH stimulation test. Baseline characteristics and outcomes of the 150 patients are shown in Table 1. Presenting symptoms included headaches (*n*=130) and scalp tenderness (*n*=75). Ischemic symptoms led to an initial GC dosage of 1 mg/kg/day (*n*=48). The mean starting dosage of GCs was 51±16 mg/day.

The mean duration of GC treatment until the first ACTH test was 17.1 months (range: 5–71 months) and the mean cumulative dose of GCs was 7.7 g (range: 2.6–22.1 g). During the follow-up, 90 patients (60%) had at least one GC-induced side effect.

### Time until adrenal function recovered

**Figure 1:**
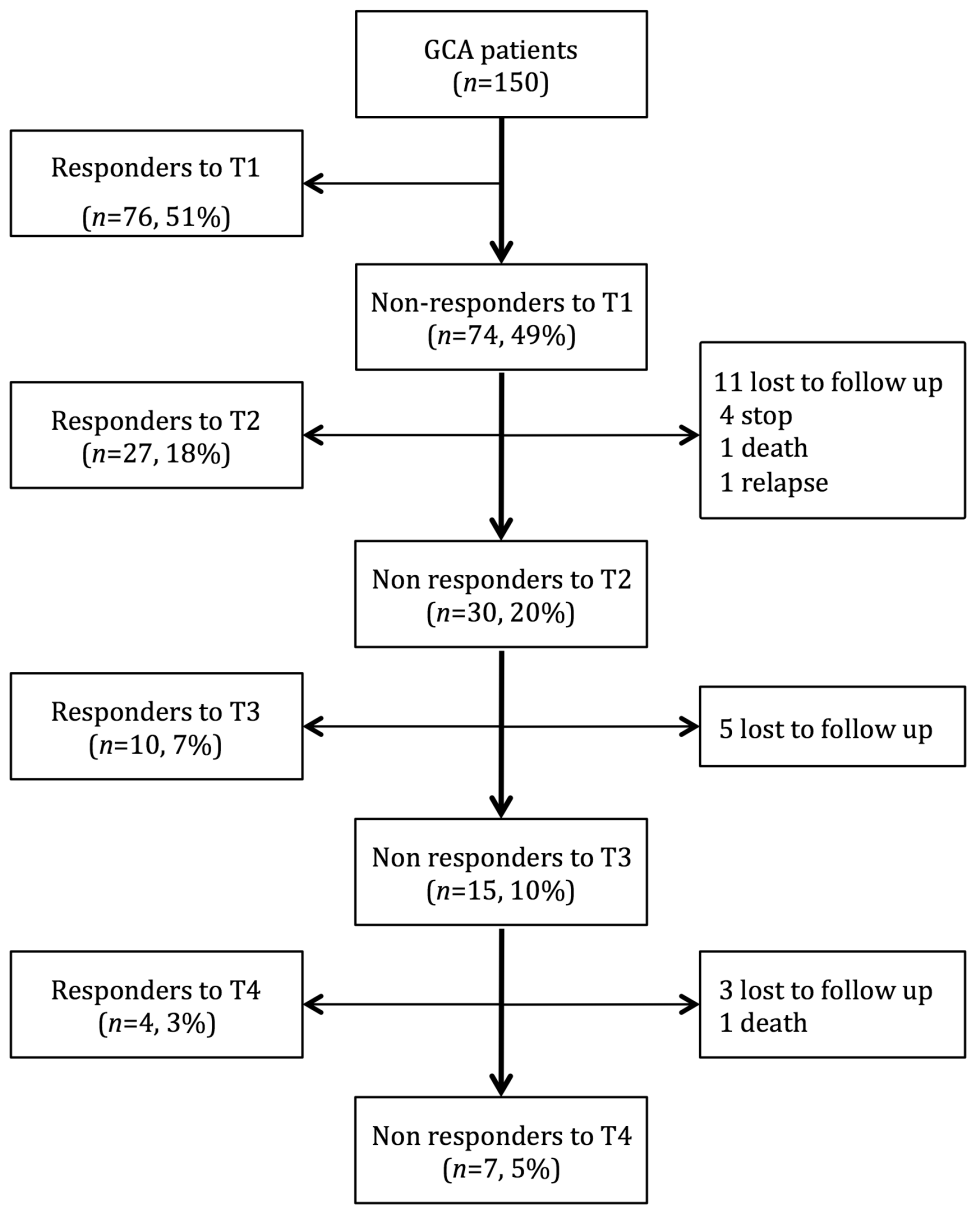
Flow chart of the study population. GCA: giant cell arteritis, T1 to T4: ACTH tests 1–4.

Seventy-four (49%) non-responders to the first ACTH stimulation test were re-evaluated annually until they had fully recovered. The patients’ outcomes are shown in Figure 1. Nineteen patients were lost to follow-up during the study, four patients voluntarily stopped the hormone substitution, two patients died (unrelated to GCA) and one patient relapsed.

Among the non-responders when first tested, 30/57 (53%) had not recovered normal adrenal function after 1 year. After 2 years (at test 3), 15 of these patients had still not recovered. By year three (at test 4), 4 patients were responders whereas 7 were not. Among patients with a negative first ACTH test, recovery of adrenal function occurred in a mean time of 14 months; the maximal time until recovery was 51 months, and this patient needed five tests. Until 36 months, 41/48 (85%) patients, who were non-responders at the first ACTH test, had recovered. Regarding the entire study population, seven patients (5%) never responded to ACTH stimulation and were finally categorized as having definitive adrenal insufficiency. Only, three of these patients received a metyrapone test, which confirmed this status.

### Risk factors for adrenal insufficiency after one year of GC therapy

Table 1 shows comparisons between responders and non-responders at the first ACTH test (prednisone dosage=5 mg/day). Age did not affect the patients’ response to this test, and no baseline characteristics varied significantly between responders and non-responders.

Basal cortisol concentrations were significantly lower in patients who did not respond to the first ACTH test. Daily GC dosage at 3, 6 and 12 months, treatment duration, and cumulative dose were significantly different (all were higher in non-responders). The occurrence of GC-associated adverse events did not differ between the responders and non-responders. There was no difference in the relapse rate between responders and non-responders to the first ACTH test (52% vs 48%, *p*=0.7).

We then transferred the continuous quantitative data to their qualitative counterparts. For GC treatment at 6 months, at 12 months, for treatment duration, and cumulative GC dose, the calculated thresholds were 15 mg/day, 9.5 mg/day, 19 months and 8.5 g, respectively.

In Table 2, univariate analyses show there was a significantly increased risk of a negative response for each parameter for non-responders, with odds-ratios (OR) ranging from 1.98 to 4.0. A basal cortisol concentration of <386 nmol/L significantly increased the risk of being a non-responder (OR=7.62, CI95% 3.7–15.8). Multivariate analyses confirmed this independent significant association.

**Table 2:** Univariate analyses of glucocorticoid (GC) dose and duration in relation to adrenal insufficiency at the first ACTH stimulation.

**Treatment**	**Responders (*n*=76)**	**Non-responders (*n*=74)**	**Odds ratio (95% confidence interval)**	***P***
**GC dosage at M6 (mg/day)**				
<15	55	43		
≥15	20	31	1.98 (1-3.9)	0.05
**GC dosage at M12 (mg/day)**				
<9.5	66	46		
≥9.5	10	28	4.0 (1.8-9.1)	0.0005
**Time from diagnosis to the first ACTH test (months)**				
<19	60	43		
≥19	16	31	2.7 (1.3-5.5)	0.006
**Total amount of GCs received until the first ACTH test (mg)**				
<8500	63	41		
≥8500	13	33	3.9 (1.8-8.3)	0.0003
Basal cortisol concentration (nmol/L)*				
≥386	58	22		
<386	18	52	7.62 (3.7-15.8)	<0.0001

M6 = after 6 months, M12 = after 12 months

* Multivariate analyses were adjusted to the basal cortisol concentration, if the total amount of GCs received until the first ACTH test was ≥8.5g, if the time from diagnosis to the first ACTH test was ≥19 months, GC dosage at M6 was ≥15 mg/d, GC dosage at M12 was ≥9.5 mg/day, gender, constitutional symptoms, fatigue, and pulsed methylprednisolone.

Finally, the relapse rate was significantly increased in the subgroup of patients who had had treatment duration of ≥19 months (OR=2.9, CI95% 1.35–6.47, *p*=0.005). The same trend was observed but did not raise statistical significance for total GC dose of ≥8.5 g. Moreover, in non-responders to the first ACTH test, the relapse rate was significantly higher when treatment duration reached ≥19 months (OR=3.7, CI95% 1.29–11.2, *p*=0.009).

## Discussion

In this study, we found that 74/150 (49%) patients treated long term with GC for GCA did not respond to the first ACTH stimulation test. The main predictive factors associated with an abnormal response were total dose and duration of GC treatment (>8.5 g and >19 months, respectively). Our results also indicate that increased treatment duration is at least partially due to the increased occurrence of relapses. A basal cortisol concentration of <386 nmol/L was strongly associated with a negative ACTH test. Also, the annual repetition of an ACTH test until 36 months allowed us to rule out adrenal insufficiency in 41/48 (85%) patients who had been first diagnosed with adrenal dysfunction.

Previous studies on long-term treatment with GC report that 15–100% of patients have adrenal insufficiency when detected by an ACTH test during final GC tapering [6]. No previous study has reported this in patients with GCA. Thus, our study is the largest to describe secondary adrenal insufficiency in older patients with rheumatic disease. The strength of our study lies in its large and homogeneous recruitment, the standardized treatment procedure, and the limited number of patients lost to follow-up.

None of our patients had an adrenal crisis. However, our patients were given hormone substitution and were strictly followed up.

Although the risk of developing such a severe complication has been known since the 1950s, its real frequency remains controversial [11,12]. One could explain the very low frequency of adrenal crisis by its central origin: the glucocorticoid-induced negative feedback to the hypothalamic and pituitary glands may result in an adrenal crisis, but usually only if there is acute physiological stress (e.g. surgical or infectious stress). Thus, given the rarity of an adrenal crisis, systematic ACTH testing does not seem to be justified. However, symptoms of slow adrenal insufficiency (e.g. weakness, fatigue, myalgia, arthralgia, depression) should not be minimized. Thus, detecting slow adrenal insufficiency may be critical because of its considerable impact on quality of life and the potential risk of disability.

Our study confirmed that total dose and duration of GC were predictive factors [8,11,13]. We determined the thresholds of 6 and 12 months of therapy, which may help physicians to evaluate an increased risk of adrenal insufficiency.

One limitation of our study may be the age- or inflammation-related changes to the HPA axis. However, previous studies have reported no difference in the response of the HPA axis between young and older subjects [14,15]. Also, compared to age-matched healthy controls, untreated GCA patients have higher cortisol levels after the ACTH test [10]. Finally, inflammatory markers (i.e., erythrocyte sedimentation rate and C-reactive protein) were negative in all patients at the time of the first ACTH test, which tends to eliminate the potential effect of inflammation on dysfunction of the HPA axis. Another limitation could be the use of the low-dose (1 µg) ACTH test, that some authors may consider less conclusive than the 250 µg standard test, whereas other state it is more sensitive. This controversy has been lasting since the value of the low-dose test was reported by Dickstein et al. [16]. Recently, two meta-analyses have been performed to try to settle this question. The first one, by Dorin et al., has reported similar operating characteristics for the low-dose and standard-dose tests, while the meta-analysis by Kazlauskaite et al. has demonstrated that low-dose test was superior to the standard test [7,17]. Both studies included some patients with corticosteroid therapy. Overall, none of the standard or the low-dose test is able to detect the entire HPA axis dysfunction. Nevertheless, these tests are the most convenient for daily practice as the insulin hypoglycaemia test is not recommended in the elderly because of dangerousness and metyrapone test can have major side effects [18]. We analysed 20 GCA patients, out of the 100 patients with rheumatic diseases who had a standard (250 µg) ACTH test, from the previous study by Pugnet et al. [19]. In this study, patients were treated according to an identical GC protocol and tested for adrenal response when the prednisone dosage was of 5 mg/day. Pugnet’s study population was similar to ours (mean age 75±7 years; 14/20 females [70%]). Mean time from the diagnosis to the first ACTH test was 21 months (range 5-47) and total GC dose was not available. We found a similar response rate at first ACTH test (9/20 responders). After a first negative test, the mean time until recovery of adrenal function was 11 months (range 3-36), similar to that of our study. Comparison between responders and non-responders for the cut-offs that we have established was not significant because of the small effective of Dr Pugnet’s study. These data suggest that the accuracies of standard and low-dose ACTH tests are similar for the detection of secondary adrenal insufficiency in the population of patients with GCA.

### Clinical application

Considering that i) an adrenal crisis is rare; ii) that most physicians and rheumatologists are aware of the long-term adverse events associated with GC therapy; and iii) that the 2009 EULAR recommendations and the 2010 BSR and BHPR guidelines for the management of GCA do not mention ACTH testing; we cannot recommend systematically performing this test during withdrawal of GCs. Nevertheless, we strongly suggest that physicians’ vigilance should be increased in this older population, especially when GC therapy is greater than the previously mentioned thresholds. For patients at risk, a safe and reasonable way to proceed could be to taper GCs more slowly and to perform a first ACTH stimulation test before GC withdrawal. If this is abnormal, a second test should be performed after 12 and 24 months (if needed).

In conclusion, we report here, for the first time, the high frequency of adrenal insufficiency after long-term GC-treatment in patients with GCA. Higher doses of GC given for a longer duration were associated with increased adrenal insufficiency. Although this complication was often transitory, a non-negligible number of patients retained adrenal insufficiency.
