# Correction: Recovery of Adrenal Function after Long-Term Glucocorticoid Therapy for Giant Cell Arteritis: A Cohort Study

**DOI:** 10.1371/annotation/66da2350-2981-489a-932d-3a456c8d0e49

**Published:** 2013-12-20

**Authors:** Yvan Jamilloux, Eric Liozon, Gregory Pugnet, Sylvie Nadalon, Kim Heang Ly, Stephanie Dumonteil, Guillaume Gondran, Anne-Laure Fauchais, Elisabeth Vidal

The name of the fifth author is incorrectly represented in the Citation. The correct Citation is: Jamilloux Y, Liozon E, Pugnet G, Nadalon S, Ly KH, et al. (2013) Recovery of Adrenal Function after Long-Term Glucocorticoid Therapy for Giant Cell Arteritis: A Cohort Study. PLoS ONE 8(7): e68713. doi:10.1371/journal.pone.0068713. 

